# Mold–Slug Interfacial Heat Transfer Characteristics of Different Coating Thicknesses: Effects on Slug Temperature and Microstructure in Swirled Enthalpy Equilibration Device Process

**DOI:** 10.3390/ma12111836

**Published:** 2019-06-06

**Authors:** Min Luo, Daquan Li, Wenying Qu, Xiaogang Hu, Qiang Zhu, Jianzhong Fan

**Affiliations:** 1 General Research Institute for Nonferrous Metals, Beijing 101407, China; lm_grinm@163.com (M.L.); quwenying2008@163.com (W.Q.); jzfan@grinm.com (J.F.); 2 Department of Mechanical and Energy Engineering, Southern University of Science and Technology, Shenzhen 518055, China; huxg@sustc.edu.cn (X.H.); zhuq@sustc.edu.cn (Q.Z.)

**Keywords:** temperature, microstructure, semi-solid slug, interfacial heat transfer coefficient, coating, rheocasting

## Abstract

Application of a coating on a mold surface is widely used in the foundry industry. Changes in coating change the heat transfer at the mold–melt interface, which influences the microstructure of the casting. In this study, the effect of boron nitride coating thickness on the interfacial heat transfer and slug microstructure in the Swirled Enthalpy Equilibration Device (SEED) process was investigated. The temperatures of the semi-solid slug and mold were measured, and the interfacial heat transfer coefficient and heat flux of the mold–slug interface was estimated based on these data. Microstructures of the quenched slugs were also examined. The results indicated that the interfacial heat transfer coefficient decreased with an increase in coating thickness and was sensitive to a coating thickness of less than 0.1 mm. The interfacial heat flux decreased sharply at the early stage, and then slowed down as the swirling time increased and the coating thickened. The coating thickness affected the temperature evolution of the slug at the early stage of the SEED process. As the coating thickness increased from near zero to 1.0 mm, the grain size of the slug increased by ~20 µm and the globular structure of the slug transformed into a dendritic structure.

## 1. Introduction

Aluminum parts, produced by efficient and low-cost casting processes, are widely used in transportation applications. Well-known casting processes include sand casting, permanent casting, and high-pressure die casting. All of these manufacturing routes have certain drawbacks, including porosity, air holes, and misruns of the casting. Semi-solid die casting, in which a semi-solid slug or slurry with fine and globular primary α-Al particles is pushed into the die cavity, was developed to eliminate these casting defects. For semi-solid die casting, preparation of the semi-solid slug is a key step. Dozens of processes have been developed to prepare such slugs with fine and globular primary α-Al particles. These processes can be classified into two categories: rheo routes and thixo routes. The rheo route has been widely adopted due to its low operating cost and high efficiency.

**Figure 1:**
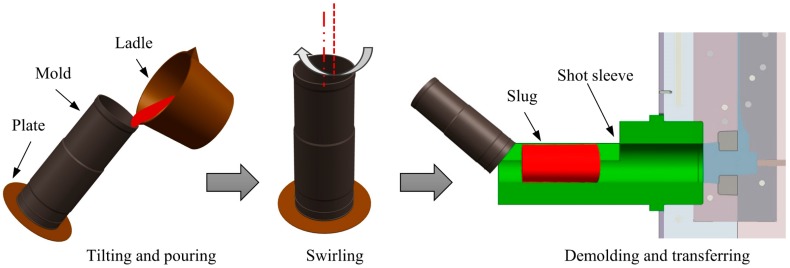
Schematic of the Swirled Enthalpy Equilibration Device (SEED) process. Adapted from Côté et al. [2].

The Swirled Enthalpy Equilibration Device (SEED) process is one such rheo route. The original version was developed by Doutre et al. from Alcan [1], and then subsequently improved by Côté et al. [2]. Figure 1 shows a diagram of this process. It is widely commercialized for semi-solid die casting due to its relative simplicity and satisfactory repeatability. To prevent the metal from sticking to the mold and to protect the mold from erosion during the SEED process, a boron nitride (BN) coating is sprayed on its internal surface. This coating changes the heat transfer between the mold and slug, and can therefore affect the microstructure of the slug. On the other hand, Nafisi et al. [3] investigated the rheological behavior of the semi-solid slug made by the SEED process by a compression test, and da Silva et al. [4] studied the rheological behavior by a cutting test. They found that the microstructure of a semi-solid slug strongly affects its rheological behavior. Liang et al. [5] and Nafisi [6] found the use of grain refiners resulted in fine structure. Liang et al. [7] and Lashkari et al. [8] found low pouring temperature was beneficial for the formation of fine microstructure. No published literature to date has shown the effect of the coating on the slug microstructure.

Interfacial heat transfer at the melt–mold interface has been well researched in sand and permanent casting. Published research has shown that many factors affect the interfacial heat transfer coefficient (IHTC). Bouchard et al. [9] found that a grooved or sandblasted mold surface enhanced heat transfer at the early stage of solidification. Muojekwu et al. [10] found that reducing the thermal resistance of the mold material, increasing superheating, and reducing the mold surface roughness was beneficial to increasing the IHTC and resulted in a finer microstructure. Hamasaiid et al. [11] found that the coating thickness on the mold affected the IHTC of the mold–melt interface and that the maximum value of the IHTC was sensitive to coating thickness for thin-layer coatings (<20 µm). With respect to the SEED process, Colbert and Bouchard [12] investigated several factors pertaining to the IHTC using orthogonal experimentation, and found that coating thickness had no obvious effect on the IHTC; however, these researchers only examined thicknesses of 30 and 60 µm. Practically, the coating thickness is influenced by the operating ability of workers; the coating also becomes thinner and thinner as the number of production cycles increases. These factors can cause the coating thickness to vary within a wide range. It is therefore meaningful to investigate the effects of coating thickness (across a relatively wide range) on the IHTC of the mold–slug interface and the microstructure of the slug. Determination of the IHTC of the mold–slug interface is also important for modeling solidification of the SEED process.

In the present study, the temperatures of the slug and mold were measured for different coating thicknesses on the internal surface of the mold. The IHTC and heat flux on the slug–mold interface were estimated according to the temperature data. The temperature fields and microstructures of slugs produced with different thicknesses of coatings were characterized and analyzed.

## 2. Materials and Methods 

Commercial aluminum alloy 357 (Al-7.3Si-0.6Mg, mass%) was melted in an electric resistance furnace and held at a temperature of 720 °C. Approximately 1.55 kg of melt at a temperature of 630 °C was poured into a cool tilted cylindrical steel mold of 78 mm inside diameter, 2.3 mm wall thickness, and 210 mm height. Eccentric rotation was then applied at a frequency of 180 rpm. Figure 1 shows a schematic of this process. The slug was removed from the mold after swirling for 180 s and immediately quenched in cold water.

**Figure 2:**
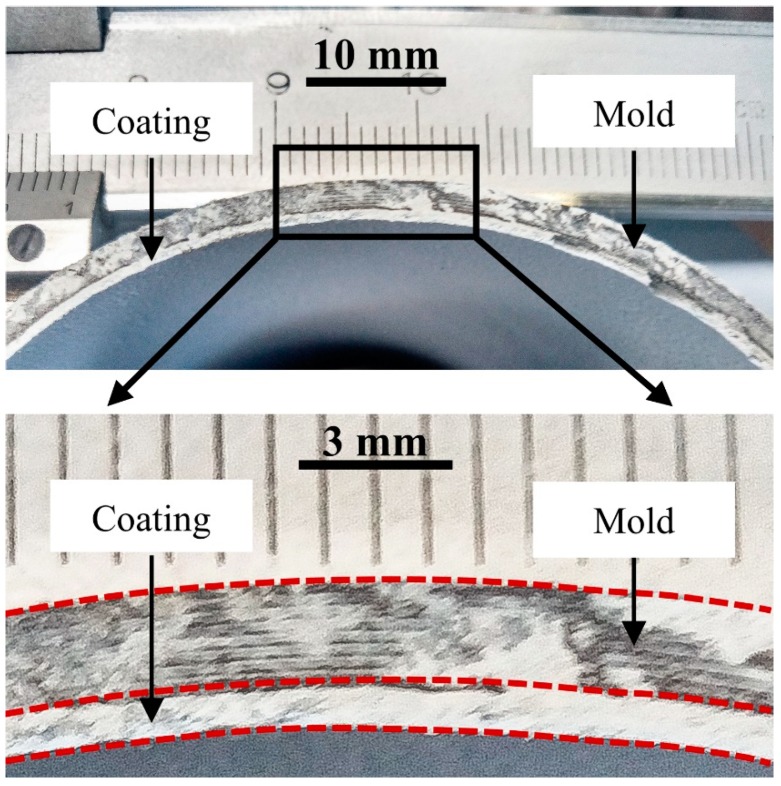
Photograph of boron nitride (BN) coating (~1 mm) on internal mold surface. The boundary of the mold and coating is marked by the red dashed line.

In the present study, four thicknesses of BN coating (provided by Pyrotek^®^ Co., Ltd., Spokane, WA, USA) were applied. The thickness of the coating was controlled by adjusting the spray time. The detailed parameters are summarized in Table 1. Figure 2 shows a photograph of a 1 mm BN coating.

**Figure 3:**
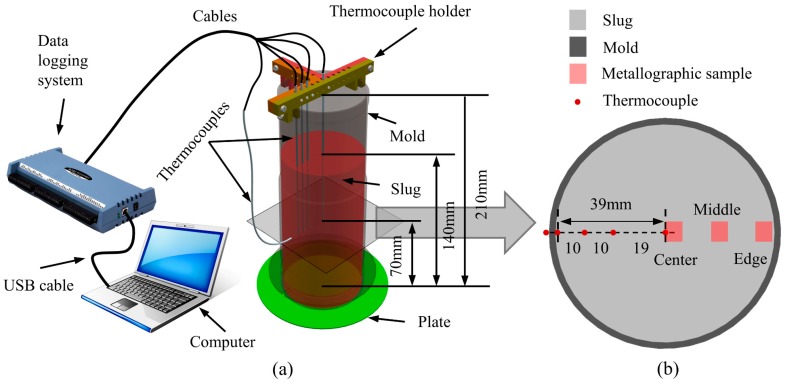
(**a**) Diagram of temperature acquisition system. (**b**) Distribution of thermocouples and metallographic samples in the radial direction.

A temperature acquisition system (see Figure 3) was designed to record the temperatures of the slug and mold from the pouring process until swirling stopped. In this system, four 2 mm diameter K-type thermocouples (measurement accuracy of about ±0.5 °C) were used to measure slug temperatures between the edge and center, and a K-type surface thermocouple (measurement accuracy of approximately ±2 °C) was used to measure the temperature of the mold. The locations of thermocouples are shown in Figure 3.

Metallographic examinations were performed at the edge, middle, and center of the quenched semi-solid slugs, as shown in Figure 3b. Standard grinding and polishing process were carried out and the samples then anodized with Barker’s reagent (5 mL HBF_4_ + 200 mL H_2_O) under 20 V direct current for 90 s at room temperature. The anodized samples were observed using an Axiovert 200 MAT microscope (Carl Zeiss, Oberkochen, Germany) using polarized light. Five micrographs (total area of 24 mm^2^) were randomly selected from each sample for quantitative analysis. Particle size (d) and shape factor (F) were evaluated using Image-Pro Plus software (Media Cybernetics, Inc., Rockville, MD, USA). The particle size of each primary particle was defined as the diameter of the equivalent circle. According to the particle area data obtained from image analysis, the size of each particle was given by

(1) $$d=2\sqrt{\frac{A}{\pi }},$$

where *A* is the area of each primary α-Al particle in the micrograph.

The shape factor (*F*) is defined as

(2) $$F=\frac{4\pi A}{P^{2}},$$

where *P* is the perimeter of the particle. For *F* = 1, the particle is a perfect circle; a particle with a more complex shape will correspond to a smaller value of *F*.

The area weighted-average shape factor (given by Equation (3)) was used to describe the average shape factor of the particles:

(3) $$F_{average}=\overset{n}{\underset{i=1}{\sum }}\left(F_{i}\frac{A_{i}}{\sum_{i=1}^{n}A_{i}}\right),$$

where *F_average_* is the average shape factor of particles in the selected region, *i* is the number of the particle, and *n* is the total number of particles in the selected region.

## 3. Results and Discussion

### 3.1. Interfacial Heat Transfer Coefficient and Heat Flux

**Figure 4:**
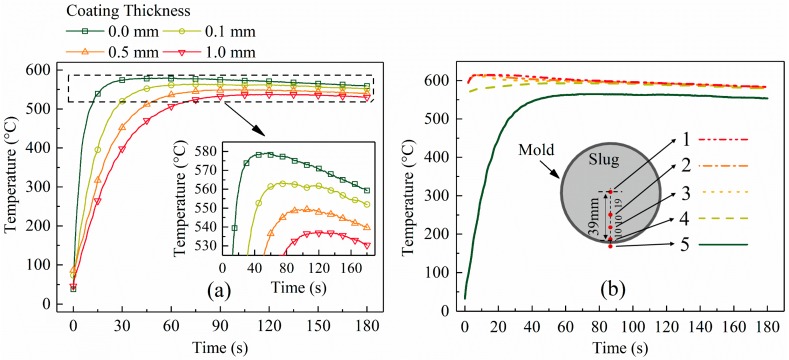
(**a**) Temperature evolution of external surface of the mold for different BN coating thicknesses; (**b**) temperature evolution of mold and slug (coating thickness: ~0.1 mm).

Figure 4a presents the temperature evolution of the mold. All curves showed several common features: (1) a sharp rise in the mold temperature at the early stage due to the large temperature difference between the mold and slug; (2) a decrease in the heating rate with increasing time due to the decreasing driving force (temperature difference between the mold and slug) until the temperature reached a maximum value; (3) air cooling of the mold at a slow and almost constant rate; and (4) a lower temperature of the mold than the slug and a nearly constant temperature difference at the later stage (see Figure 4b). As the coating thickness increased, the heating rate of the mold at the early stage decreased and the maximum temperature of the mold reduced. This was attributed to changes in the IHTC and heat flux on the mold–slug interface due to the change in coating. These parameters were quantitatively analyzed based on the temperature data.

In the SEED process, the heat released by the melt is absorbed by the mold and ambient air, so the heat flux (q) (W·m^−2^) on the mold–slug interface can be calculated by

(4) $$q=\frac{dT}{dt}\frac{V}{A}\rho c+h_{ma}(T_{me}-T_{a}),$$

where $V/A=\left(r_{mold}^{2}-r_{slug}^{2}\right)/\left(2r_{slug}\right)$ ($r_{slug}$ and $r_{mold}$ are the radii of the mold and slug, respectively); T is the instant average temperature of the mold (°C); t is the time (s); ρ is the density of the mold material (kg·m^−3^); c is the specific heat of the mold material (J·kg^−1^·°C^−1^); $h_{ma}$ is the IHTC of the mold–air interface (W·m^−2^·K^−1^); $T_{me}$ is the temperature of the external mold surface (°C); and $T_{a}$ is the air temperature (°C). On the right of Equation (4), the first component is the heat flux caused by the mold and the second component is that caused by the air.

**Figure 5:**
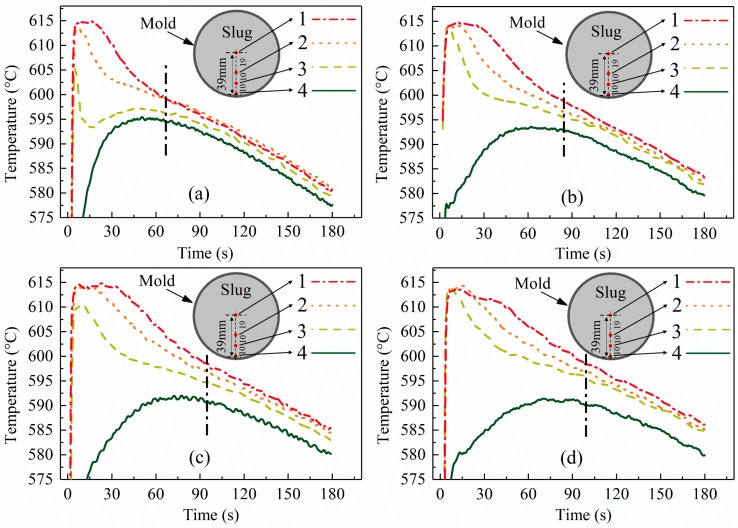
Temperature evolution of slug under different thicknesses of BN coating: (**a**) 0.0 mm, (**b**) 0.1 mm, (**c**) 0.5 mm, and (**d**) 1.0 mm. The black dashed lines mark the times at which the slug temperature distribution changed from unstable to quasi-stable.

According to Newton’s law of cooling, the IHTC of the mold–slug interface (*h*) (W·m^−2^·K^−1^) can be calculated by

(5) $$h=\frac{q}{T_{ss}-T_{mi}},$$

where $T_{ss}$ is the temperature of the slug surface (Figure 5**,** location 4) (°C) and $T_{mi}$ is the temperature of the internal mold surface (°C).

In this study, the temperature gradient within the mold could be ignored, so T and $T_{mi}$ were represented by the temperature of the external mold surface ($T_{me}$) (see Figure 4a). The reason is that the Biot number (Bi=hl/λ) was ~0.02–0.22 (where h was ~300–3000 W·m^−2^·K^−1^; l is the wall thickness of the mold, 0.0023 m; λ is the thermal conductivity of mold, ~30 W·m^−1^·K^−1^). According to the heat transfer literature of Lienhard IV and Lienhard v [13], the temperature gradient in an object can be ignored when Bi≪1; for example, the temperature gradient in thermocouple.

**Figure 6:**
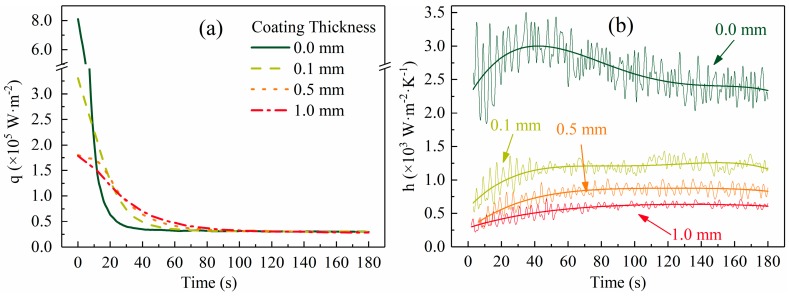
(**a**) Interfacial heat flux evolution for different coating thicknesses and (**b**) estimated interfacial heat transfer coefficient for different coating thicknesses. The high frequency waves of the curves resulted from the measurement error.

According to Equations (4) and (5)**,** the parameters in Table 2, the mold temperature data (Figure 4a), and the slug surface temperature (Figure 5, location 4), the heat flux and IHTC were calculated and are plotted in Figure 6a,b, respectively.

Figure 6a shows that the largest heat flux appeared at the beginning and then decreased sharply with time until a nearly constant value of 30,000 W·m^−2^ was reached. As the coating thickness increased, the largest heat flux decreased and its rate of reduction slowed down. The largest heat flux decreased from 800,000 W·m^−2^ to 180,000 W·m^−2^ when the coating thickness increased from 0.0 mm to 1.0 mm.

Figure 6b shows that the IHTC decreased with increase in coating thickness. The IHTC sharply decreased by more than 50% when the coating thickness increased from 0.0 mm to 0.1 mm. This result is similar to that of Hamasaiid et al. [11], who found the IHTC was sensitive to coating thickness when the coating was very thin. Unlike other casting processes, however, all IHTC curves fluctuated within a relatively small range during the SEED process. With respect to other casting processes, Sun et al. [15] found that the IHTC decreased from ~6000 W·m^−2^·K^−1^ to ~800 W·m^−2^·K^−1^ during squeeze casting; Lau et al. [16] showed that the IHTC decreased sharply from ~2000 W·m^−2^·K^−1^ to ~500 W·m^−2^·K^−1^ during permanent mold casting; Zhi-peng et al. [17] found that the IHTC was reduced by more than 75% in high-pressure die casting. Reduction of the IHTC is caused by the increase of the air gap between the casting and mold in these processes. In the SEED process, the semi-solid slug is soft enough to avoid the formation or increase of the air gap.

### 3.2. Temperature Distribution and Evolution of Semi-Solid Slug

**Figure 7:**
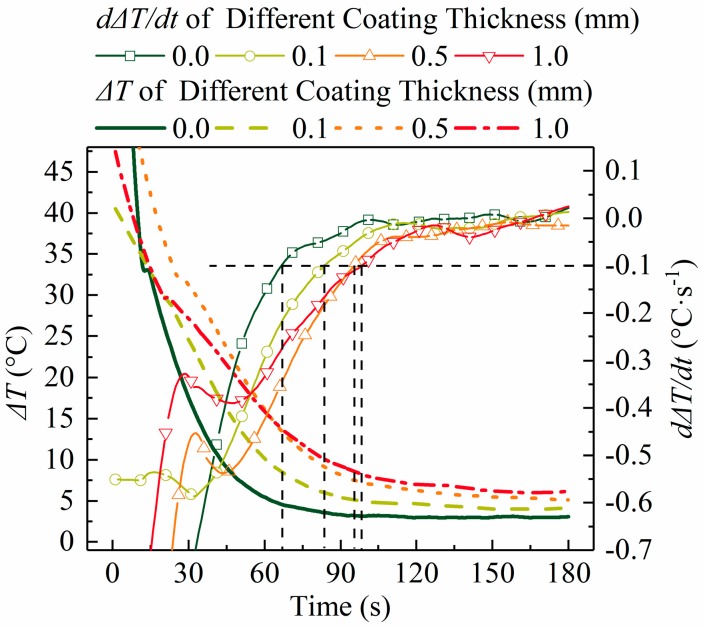
Temperature differences between slug center (Figure 5, point 1) and edge (Figure 5, point 4) and its differential with time. The dashed lines mark the end of the unstable stage.

**Figure 8:**
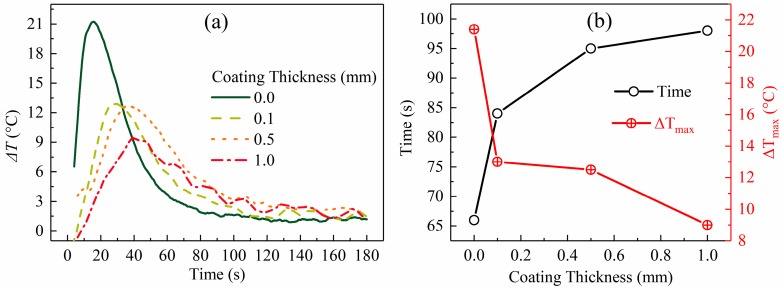
(**a**) Evolution of temperature difference between points 1 and 3 in Figure 5 for different coating thicknesses. (**b**) Duration of unstable stage and maximum temperature difference between points 1 and 3 in Figure 5 for different coating thicknesses.

Figure 5, Figure 7, and Figure 8a show that a large temperature gradient (or temperature difference) was built in the radial direction of the slug at the beginning of the process and then decreased as the swirling time increased until a nearly constant value was reached. These results are supported by the interfacial heat flux data shown in Figure 6a. According to Fourier’s law, the temperature gradient is proportional to heat flux: Figure 6a shows that the interfacial heat flux presented a similar trend (decreased from a very high value to a nearly constant low value).

According to the characteristics of these curves, the temperature evolution of the slug could be divided into two stages: unstable and quasi-stable stages. A large and unstable temperature gradient existed in the slug during the unstable stage; the temperature gradient was stable and small during the quasi-stable stage and the cooling rate of the entire slug was almost the same and constant. The critical time was defined as the time at which the temperature difference between the slug center (Figure 5, point 1) and edge (Figure 5, point 4) was equal to −0.1 °C·s^−1^ (see Figure 7, marked using dashed lines).

The temperature curves of the slugs were impacted by the variation in coating thickness. These influences are summarized and discussed in the following points.

1. The duration of the unstable stage became shorter as the coating thickness reduced (see Figure 8b). These results are coincident with those of heat flux shown in Figure 6a. Figure 6a shows that, for a slug with a thinner coating, less time is required to reduce the heat flux to a nearly constant low value.
2. Figure 5 shows that the highest temperature gradient decreased with an increase in coating thickness. To show this clearly, the largest temperature difference between points 1 and 3 in Figure 5 was used to represent the largest temperature gradient and is plotted in Figure 8b. Figure 8b shows that this temperature difference decreased from 21 °C to 9 °C as the coating thickness increased. As shown in Figure 6b, the IHTC reduced from ~2700 W·m^−2^·K^−1^ to ~500 W·m^−2^·K^−1^ when the coating increased from 0.0 mm to 1.0 mm, which caused the highest heat flux to decrease from 800,000 W·m^−2^ to 180,000 W·m^−2^, respectively (see Figure 6a). This is the reason for the variation of this temperature gradient.
3. The final temperature (Figure 5, location 1 at 180 s) decreased from 586 °C to 581 °C with the decrease in coating thickness. This observation is supported by the temperature data of the mold (see Figure 4a): a mold with a thick coating exhibited a lower temperature than a mold with a thin coating. Less heat was absorbed by the mold with a thick coating and the rate of heat loss to the air also reduced.
4. As the coating thickness reduced, the temperature difference between the slug center and surface during the quasi-stable stage decreased (see Figure 5 and Figure 7). Figure 8a shows that the temperature difference between slug center and middle was almost same for various coating thicknesses. The increasement in Figure 7 could result from measurement error: the thermocouple at the slug surface touched the mold during the experiments, which may have resulted in the experimental values being slightly lower than the true values. The influence of this effect would have increased as the mold temperature reduced (Figure 4a shows that the mold temperature reduced as the coating thickened). It was concluded that the coating thickness had no obvious effect on the radial temperature distribution of the slug during the quasi-stable stage. This conclusion is supported by the data of Figure 6a, which shows that the interfacial heat flux of different coating thicknesses was almost same during the quasi-stable stage.

### 3.3. Microstructures of Different Coating Thicknesses

**Figure 9:**
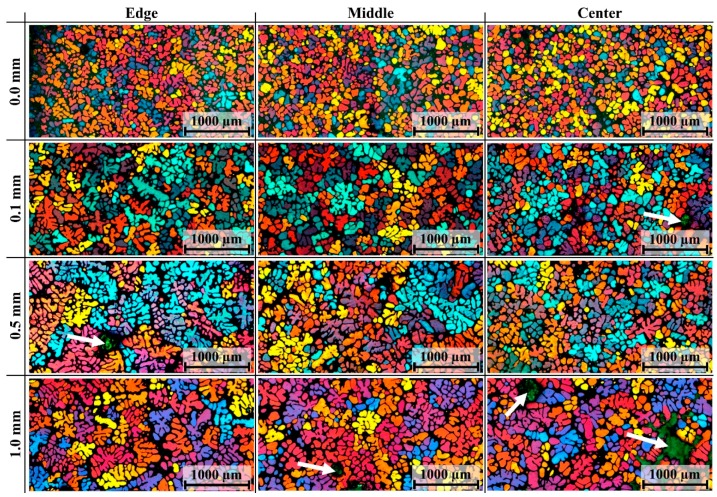
Representative micrographs of quenched slug under different thicknesses of boron nitride coating. The bottle green region marked by white arrows is porosity which formed during the quench process. (The color information of this figure is shown in the web or color print version of this paper.)

**Figure 10:**
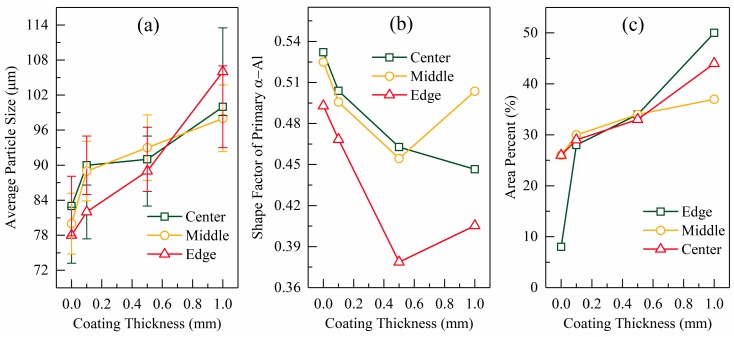
Relationship between coating thickness and (**a**) particle diameter, (**b**) average shape factor, and (**c**) area percentages of grains with sizes larger than 170 µm.

Typical microstructures of the quenched semi-solid slugs obtained for different coating thicknesses are presented in Figure 9. As the coating thickness diminished, the grain size obviously decreased and the dendritic structure transformed to globular and rosette-like structures. These conclusions are supported by quantitative analysis. The data show that when the coating thickness increased from 0.0 mm to 1.0 mm, the grain size enlarged from 78 µm to 106 µm at the edge of the slug, from 80 µm to 98 µm at the middle, and from 83 µm to 100 µm at the center (see Figure 10a). The area percentage of large particles (*d* ≥ 170 μm) increased as the coating thickened (see Figure 10c). Figure 10b shows an overall trend of a decreasing average shape factor as the coating thickened. There were also more dendritic structures at the edge than at the center, which is supported by the quantitative analysis presented in Figure 10b.

According to the fundamentals of solidification, both nucleation and growth determine the morphology and size of a solidified grain. A high nucleation rate and slow cooling rate (slow growth rate) usually impede the growth of a dendritic microstructure and result in fine structure. This conclusion has been recognized by most researchers in this area. Zhu et al. [18] investigated the evolution of globular and dendritic microstructures of an Al-7mass%Si alloy using a modified cellular automaton model and found that a high nucleation rate combined with a low cooling rate was beneficial to the formation of a globular structure. Uggowitzer and Kaufmann [19] reached this conclusion experimentally. The SEED process combines these two aspects by pouring a low superheated melt into a well-designed mold. During the pouring process (see Figure 1) and the unstable stage many nuclei are generated in the deep undercooled melt caused by the mold; a slow cooling rate (see Figure 5) then allows these grains to grow slowly.

In the present study, the observation that a thick coating resulted in a large dendritic structure can be explained by nucleation and grain growth. The analysis in Section 3.1 showed that a 1.0 mm coating reduced both the heat flux during the first 10 s and the IHTC to approximately one-fifth of the respective values for a 0.0 mm coating (see Figure 6). The cooling rate of the melt during the pouring process and early period of unstable stage therefore decreased sharply in the case of a thicker coating, which resulted in small undercooling and a low nucleation rate. With respect to growth, a thick coating was also beneficial to the growth of a dendritic structure. Normally, both nucleation and growth occurred during the unstable stage, while nucleation mainly occurred during the pouring process and for a short period thereafter. Xu et al. [20] investigated the solidification process of Al-10mass% Cu using in situ X-radiography, and found the nucleation ceased after solidification for 20 s when the cooling rate was 0.05 K/s, and that this time decreased with an increase in cooling rate. In this study, we assumed that the nucleation ceased 20 s after pouring. Figure 4b shows that as the coating thickened, the mold temperature at 20 s decreased and the time required for the mold temperature to rise to its highest value increased. This meant that the intensity of melt cooling and the cooling time increased as the mold coating thickness increased during the later period of the unstable stage. The interfacial heat flux data (see Figure 6a) also supported this conclusion. In summary, a thicker coating reduced the nucleation rate during the pouring process and the early period of unstable stage, increased the grain growth rate during the later period of unstable stage, and extended the duration of unstable stage, and so resulted in the formation of large dendritic structures. The dendritic structures at the slug edge might have resulted from the very high cooling rate, which caused a high nucleation rate, but also increased the growth rate of the grains.

## 4. Conclusions

1. The IHTC of the mold–slug interface in the SEED process was estimated. The IHTC fluctuated within a small range and decreased with an increase in coating thickness. The IHTC reduced from ~2700 W·m^−2^·K^−1^ to ~1100 W·m^−2^·K^−1^, 800 W·m^−2^·K^−1^, and 500 W·m^−2^·K^−1^ when the coating increased in thickness from 0.0 mm to 0.1 mm, 0.5 mm, and 1.0 mm, respectively. The IHTC was sensitive to the BN coating thickness when this was less than 0.1 mm.
2. The heat flux of the mold–slug interface decreased sharply from its highest value to a nearly constant low value during the SEED process. The reduction rate decreased as the BN coating thickness and swirling time increased. The highest value decreased from ~800,000 W·m^−2^ to ~180,000 W·m^−2^ when the coating increased from 0.0 mm to 1.0 mm.
3. The temperature difference in the slug dropped off to a constant low value as the swirling time rose. According to this characteristic, the temperature evolution was divided into two stages: unstable and quasi-stable stages. As the coating thickened, the duration of the unstable stage increased and the highest temperature gradient in the slug decreased. The coating thickness had no obvious effect on the quasi-stable stage.
4. The microstructures strongly depended on the BN coating thickness. When the coating thickened, the grain size enlarged and the grain morphology transformed from a globular shape to a dendritic structure. The grain size enlarged from 78 µm to 106 µm at the edge, from 80 µm to 98 µm at the middle, and from 83 µm to 100 µm at the center when the BN coating thickness increased from 0.0 mm to 1.0 mm.

The present study showed that the coating thickness on the internal surface of a mold is very important for control of the microstructure of the semi-solid slug in the SEED process. The spray coating process should be well controlled during production to produce a thin and stable coating. In other semi-solid slug preparation methods, especially those based on extensive nucleation caused by cooling of the mold (such as the New Rheocasting (NRC) process patented by Adachi et al. [21] and the Direct Thermal Method (DTM) developed by the group of Browne et al. [22]), the thickness of the coating on the mold should also be carefully controlled.

## Figures and Tables

**Table 1:** Experimental parameters.

Experiment Number	1	2	3	4
Spray time (s)	2	15	60	120
Coating thickness (mm)	~0.0	~0.1	~0.5	~1.0

**Table 2:** Values of parameters used in this study.

Parameter	ρ	c	$T_{a}$	$h_{ma}$	$r_{mold}$	$r_{slug}$
Unit	kg·m^−3^	J·kg^−1^·°C^−1^	°C	W·m^−2^·K^−1^	m	m
Value	7850	600	30	60 ^a^	0.0413	0.0390

^a^ Refer to Qu et al. [14].
